# Supplementary material for: The structure of prevacuolar compartments in Neurospora crassa as observed with super-resolution microscopy
Source: PLoS One. 2023 Apr 24;18(4):e0282989. doi: 10.1371/journal.pone.0282989 (PMC10124863; doi:10.1371/journal.pone.0282989)
Supplement: Attachment — Submitted filename: Comments December 11 2022.docx [file pone.0282989.s008.docx]

1. Recommendation (Accept, Minor Revision, Major Revision, Reject)

Answer: Accept after minor revision.

2. Is the manuscript technically sound, and do the data support the conclusions? (Answer options: Yes, No, Partly)

Answer: Yes

3. Has the statistical analysis been performed appropriately and rigorously? (Answer options: Yes, No, I don't know, N/A)

Answer: Not applicable

4. Does the manuscript adhere to the PLOS Data Policy? (Answer options: Yes, No) Additional details can be found at <http://www.plosone.org/static/policies#sharing>

Answer: Yes

5. Is the manuscript presented in an intelligible fashion and written in standard English? (Answer options: Yes, No)

**Answer: No (minor correction is required)**

6. Review Comments to the Author (Minimum 200 characters)

Answer: The manuscript titled “The Structure of Prevacuolar Compartments in Neurospora crassa as observed with Super-Resolution Microscopy” by Barry Bowman describes very new findings about the prevacuolar compartments (PVCs) of *Neurospora crassa*. The manuscript is technically sound providing deeper insight to the PVC that appears to be rapidly changing in their structure.

The manuscript may be accepted after minor revisions as suggested below.

1. This is a single author manuscript. Therefore, “we” may be replaced with “I” in the sentences like (i) “By using super-resolution microscopy we have achieved a better understanding of the structure of the PVCs (line 21−23);

(ii) “We hypothesize that…. (line 65)”; and

(iii) “We previously reported … (line 245)” etc.

2. The reference to the figures are not consistent in the manuscript. At some places it is in the main sentence and at some other places it is in the parentheses,

e. g.

line: 138: shown in figure 1..

line 151. The image in Fig 1

line 165: (Fig 2B)…

It is better to use a consistent style, preferably referring to the figures within the parentheses as in the line 165.

3. Line 283: In our previous work we observed a possible maturation process in PVCs…

Please provide supporting reference for this line.

4. Fig. 1: Kindly insert a single space in the scale, it should be 5 μm.

7. Would you like your identity revealed to the authors of this submission? (Answer options: Yes, No)

Answer: Yes

8. Do you have any potentially competing interests? If none, type "None." Our policy on competing interests can be found at <http://www.plosone.org/static/policies.action#competing>

Answer: None

9. Do you want to get recognition for this review on Publons (<https://publons.com/benefits/reviewers/how>)? If you opt in, your Publons profile will automatically be updated to show a verified record of this review in full compliance with the journal's review policy. If you don't have a Publons profile, you will need to first create a free account. (Answer options: Yes, No)

Answer: Yes
