# Supplementary material for: The structure of prevacuolar compartments in Neurospora crassa as observed with super-resolution microscopy
Source: PLoS One. 2023 Apr 24;18(4):e0282989. doi: 10.1371/journal.pone.0282989 (PMC10124863; doi:10.1371/journal.pone.0282989)
Supplement: Attachment — Submitted filename: Response to Reviewers.docx [file pone.0282989.s009.docx]

Response to Reviewers.

I thank the reviewers for carefully reading my manuscript.

Reviewer #1. I have followed through on the suggestion to use a microtubule polymerization inhibitor. The results are shown in figure 6 and described in lines 244 - 262. The results indicate that microtubules are not required for the maintenance of PVC structure and that the aggregation of dynein associated with PVCs can also be maintained when microtubules have depolymerized.

The reviewer asks for an example (I assume an image or video) of vesicles entering and exiting a PVC. I would love to see this but it has not been technically possible, at least not with the microscopes available to me. Section 3.3 in the paper is my best attempt to do so. I see rapid changes in structure but I have not observed actual fusion. Vesicles move very quickly, and the actual cargo vesicles may be as small as 90 nm, at the limit of detection for light microscopy.

In the Discussion I say “These observations suggest a hypothesis.” I put it as a possible explanation, but I don’t claim to have demonstrated it.

L30, L296, 297, 301, I italicized the names

L154 Panel A in the figure was intentionally overexposed to make the outline of the tip more visible.

Reviewer #2. The reviewer feels that the manuscript does add scientifically significant aspects beyond what was presented in my 2015 paper. Part of the motivation for doing this high-resolution microscopy was to address questions raised by reviewers of that 2015 paper. In that paper I suggested that there was no “inside” to the PVC. With soluble internal markers the PVCs often appeared as donuts, not filled spheres. I was requested to deemphasize this conclusion because there was not sufficient visual evidence. The structural detail in my current manuscript provides a much more accurate understanding of the structure of this organelle.

L49 Forgac reference removed

L56-57 Citation of Bowman 2015 added

L164 The microscopy does not produce a clear edge for the vesicles. In the figure I show a 2 micron scale bar. The vesicles vary in size but their diameters are approximately 1/8^th^ the length of that line.

L238 The images for microtubules are in the supplementary movie. I have added Fig 6 to give more information about the role of microtubules.

Reviewer #3. This is the only single authored manuscript in my career, and it seems a little odd to say “I observed…” But the reviewer is correct. I changed “we” to “I”, except when referring to previous multi-authored papers.

To be consistent, reference to Figs are now in parentheses e.g. (Fig. 1).

L283 the citation has been added.

Fig 1 micron scale fixed.
